# EMBLmyGFF3: a converter facilitating genome annotation submission to European Nucleotide Archive

**DOI:** 10.1186/s13104-018-3686-x

**Published:** 2018-08-13

**Authors:** Martin Norling, Niclas Jareborg, Jacques Dainat

**Affiliations:** 1 National Bioinformatics Infrastructure Sweden (NBIS), SciLifeLab, Uppsala Biomedicinska Centrum (BMC), Husargatan 3, 751 23 Uppsala, Sweden; 2 IMBIM-Department of Medical Biochemistry and Microbiology, Uppsala University, Uppsala Biomedicinska Centrum (BMC), Husargatan 3, Box 582, 751 23 Uppsala, Sweden; 3 Department of Biochemistry and Biophysics, Stockholm University/SciLifeLab, Box 1031, 171 21 Solna, Sweden

**Keywords:** Annotation, Converter, Submission, EMBL, GFF3

## Abstract

**Objective:**

The state-of-the-art genome annotation tools output GFF3 format files, while this format is not accepted as submission format by the International Nucleotide Sequence Database Collaboration (INSDC) databases. Converting the GFF3 format to a format accepted by one of the three INSDC databases is a key step in the achievement of genome annotation projects. However, the flexibility existing in the GFF3 format makes this conversion task difficult to perform. Until now, no converter is able to handle any GFF3 flavour regardless of source.

**Results:**

Here we present EMBLmyGFF3, a robust universal converter from GFF3 format to EMBL format compatible with genome annotation submission to the European Nucleotide Archive. The tool uses json parameter files, which can be easily tuned by the user, allowing the mapping of corresponding vocabulary between the GFF3 format and the EMBL format. We demonstrate the conversion of GFF3 annotation files from four different commonly used annotation tools: Maker, Prokka, Augustus and Eugene.

EMBLmyGFF3 is freely available at https://github.com/NBISweden/EMBLmyGFF3.

## Introduction

Over the last 20 years, many sequence annotation tools have been developed, facilitating the production of relatively accurate annotation of a wide range of organisms in all kingdoms of the tree of life. To describe the features annotated within the genomes, the Generic Feature Format (GFF) was developed. Facing some limitation using the originally published Sanger specification, GFF has evolved into different flavours depending on the different needs of different laboratories. The Sequence Ontology Project (http://www.sequenceontology.org; [1]) in 2013 proposed the GFF3 format, which “addresses the most common extensions to GFF, while preserving backward compatibility with previous formats”. Since then, the GFF3 format has become the de facto reference format for annotations. Despite well-defined specifications, the format allows great flexibility allowing holding wide variety of information. This flexibility has resulted in the format being used by a broad range of annotation tools (e.g. MAKER [2], Augustus [3], Prokka [4], Eugene [5]), and is used by most genome browsers (e.g. ARTEMIS [6], Webapollo [7], IGV [8]). The flexibility of the GFF3 format has facilitated its spread but raises a recurrent problem of interoperability with the different tools that use it. Indeed, one can meet as many flavours of GFF3 format as tools producing it. One of the natural outcomes of a GFF3 file is to be converted in a format that can be submitted in one of the INSDC databases. Since 2016 NCBI released a beta version of a process to submit GFF3 or GTF to GenBank [9]. They describe what information is expected in the GFF3 file and how to format it in order to be accepted by the table2asn_GFF tool, which convert the well-formatted GFF3 into.sqn files for submission to GenBank. Modifying a GFF3 file to fulfil the requirements is not often an easy task and programming skills may be needed to automate it. To facilitate this step, a user-friendly bioinformatics tool called Genome Annotation Generator (GAG) has been implemented [10]. GAG provides a straightforward and consistent tool for producing a submission-ready NCBI annotation in.tbl format. This.tbl format is a tabulate format required with two other files (.sbt and.fsa) by the tbl2asn tool provided by the NCBI in order to produce a.sqn file for submission to GenBank.

While the NCBI doesn’t accept their GenBank Flat File format but rather an.sqn intermediate file for submission, EBI accepts submission in their EMBL flat file format. Here the difficulty is to generate an EMBL flat file from a GFF3 file. Several tools have been developed to perform this step i.e. Artemis [6], seqret from EMBOSS [11], GFF3toEMBL [12] but have limitations. While plethoric number of annotation tools exist, GFF3toEMBL [12] only deals with the GFF3 produced by the prokaryote annotation tool Prokka. So, for annotation produced by other tools, users have to turn to other solutions. Artemis has a graphical user interface, which doesn’t allow an automation of the process. Seqret is designed to deal with only one record at a time, which makes its use for genome-wide annotation not straightforward. The main bottleneck is that both tools need a properly formatted GFF3 containing the INSDC expected vocabulary (3th and 9th column), while the annotation tools do not necessarily use this vocabulary. The EMBL format follows the INSDC definitions and accepts 52 different feature types, whereas the GFF3 mandates the use of a Sequence Ontology term or accession number (3rd column of the GFF3), which nevertheless constitutes 2278 terms in version 2.5.3 of the Sequence Ontology. Moreover, the EMBL format accepts 98 different qualifiers, where the corresponding attribute tag types in the 9th column of the GFF3 are unlimited. Consequently, in many cases the user may have to pre-process the GFF3 to adapt it to the expected vocabulary.

The information contained and the vocabulary used in a GFF3 file can differ a lot depending of the annotation tool used. On top of that the vocabulary used by the GFF3 format and by the EMBL format can differ in many points. Those differences make it difficult to create a universal GFF3 to EMBL converter that would avoid pre-processing of the GFF3 annotation file. The challenge undoubtedly lies in the implementation of a correct mapping between the feature types described in the 3rd column, as well as the different attribute’s tags of the 9th column of the GFF3 file and the corresponding EMBL features and qualifiers.

In collaboration with the European Nucleotide Archive we have developed a tool addressing these difficulties called EMBLmyGFF3, which is a universal GFF3 to EMBL converter allowing the submission to the ENA database. To our knowledge, this is the only tool able to deal with any flavour of GFF3 file without any pre-processing of the file. Indeed, the originality lies in json mapping files allowing the mapping of vocabulary between GFF3 and EMBL formats.

## Main text

### Implementation

The EMBLmyGFF3 tool is an implementation in the python programming language of the verbose documentation provided by the European Bioinformatics Institute [13]. The implementation is structured around two main modules: feature and qualifier.

(i) The *feature* module contains the description of all the EMBL features and their associated qualifiers. The feature module handles a parameter file in json format, called translation_gff_feature_to_embl_feature.json, allowing the proper mapping of the feature types described in the 3rd column of the GFF3 file to the chosen EMBL features.

Below is an example how to map the GFF3 feature type “three_prime_UTR” to the EMBL feature type “3′UTR”:
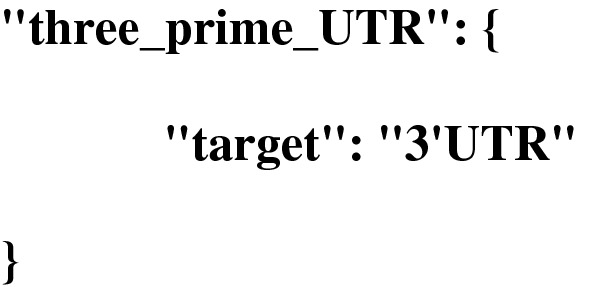


We also provide the possibility to decide which features will be printed in the output using the “remove” parameter. In the following example the feature type “three_prime_UTR” will be ignored:
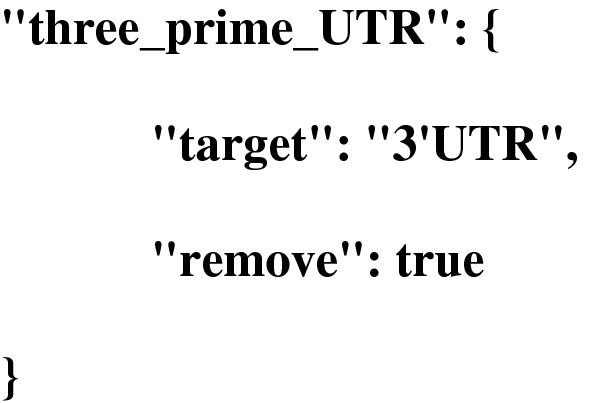


(ii) The *qualifier* module defines all the EMBL qualifiers (a definition, a comment, and the format of the expected value) and has methods to access and print them. Within the GFF3 file, the qualifiers are the attribute’s tags of the 9th column. It is common that an attribute’s tag doesn’t correspond to a EMBL qualifier name. To address this difficulty, the module handles a parameter file in json format, called translation_gff_attribute_to_embl_qualifier.json, allowing proper mapping of the attribute’s tag described in the 9th column of the GFF3 file to the chosen EMBL qualifier. Below is an example how to map the “Dbxref” attribute’s tags from the GFF3 file to the “db_xref” qualifier expected by the EMBL format.
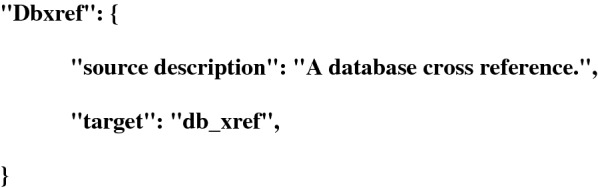


In the same way, the converter also allows the possibility to map the “source” (2nd column) as well as the “score” (6th column) from the GFF3 to an EMBL qualifier using the translation_gff_other_to_embl_qualifier.json mapping file.

Using the qualifier’s json files, we provide the possibility to add a prefix or a suffix to the attribute’s value. In the following example, if the “source” value within the GFF3 file is e.g. “Prokka”, the EMBL output will look like **note=“source:Prokka”** instead of of **note=“Prokka”**.
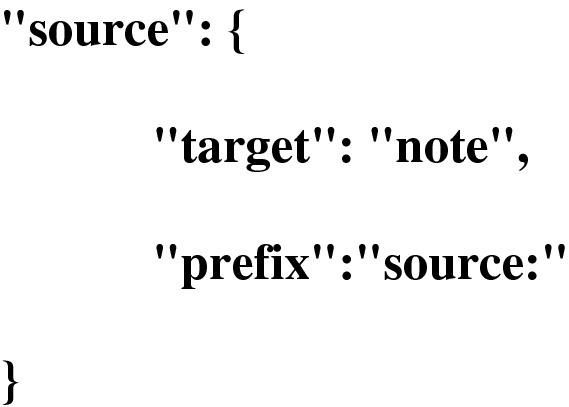


The key elements of our converter that make it universal are the parameter files in json format that describe how to map the feature types of the 3rd column, as well as the different attribute’s tags of the 9th column of the GFF3 file to the correct EMBL features and qualifiers. The json files are accessible using the –expose_translations parameter. By default, when the json parameter file doesn’t contain mapping information for a feature or qualifier, the tool checks if the name of the feature type or the tag from the GFF3 file exists within the EMBL features or qualifiers accordingly. Where relevant, the feature type or tag will be skipped, and the user will be informed, giving the possibility to add the correct mapping information in cases where this information is needed.

As requirements, the tool takes as input a GFF3 annotation file and the FASTA file that has been used to produce the annotation, as well as metadata required by ENA. There are metadata that are not contained in GFF3 format, but that are mandatory or recommended for producing a valid EMBL flat file. When all the mandatory metadata are filled, the tool will proceed to the conversion; otherwise it will help the user to fill the needed information.

### Results

The software has been used to convert the GFF3 annotation file produced by different annotation tools (e.g. Prokka, Maker, Augustus, Eugene). Three test cases are included into the source code distribution. The EMBL files produced have been successfully checked using the ENA flat file validator version 1.1.178 distributed by EMBL-EBI [14]. EMBLmyGFF3 has been also use for the submission of the annotation of two *Candida intermedia* strains performed with the genome annotation pipeline MAKER [15], as well as the annotation of *Ectocarpus subulatus* performed with Eugene [16]. The resulting EMBL files have been then deposited in the European Nucleotide Archive (ENA) and are accessible under the project accession number PRJEB14359 and PRJEB25230 respectively.

To assess the performance of our tool we have converted 5 different genomes and annotations Table 1. The computational time is tightly linked to the number of annotated features to process. In spite the slow process of huge annotation, the tool has always achieved the conversion successfully.

**Table 1:** EMBLmyGFF3 performance assessment on different genome annotations

Species	File size of the genome (Mbases)	File size of the gff annotation file (Mbytes)	Memory usage (Gbytes)	Compute time (Min)
*E. coli*	4.6	3.5	0.17	2.2
*C. intermedia*	13	3	0.14	1.8
*T. terrestris*	36	17	0.40	10
*E. subulatus*	227	63	1.60	49
*O. niloticus*	927	122	3.11	164

The tests have been run on a 2.8 GHz Intel Core i7 computer with 8 Gb memory except for *E. subulatus* run on AMD Opteron(tm) Processor 6174 with 256 Gb memory

### Discussion

Going from an annotation in one of the many existing GFF3 flavours to a correctly formatted EMBL file ready for submission is a bridge that is in most cases cumbersome and difficult to cross. We have filled this gap by developing the software EMBLmyGFF3, which has been designed to be able to easily adapt to the different GFF3 files that could be met. It successfully converts annotation files from different annotation tools and checks the integrity of the results using the official flat file validator provided by EMBL-EBI. EMBLmyGFF3 facilitates the submission of GFF3 annotations derived from any source. We hope it will help to increase the amount of data that are actually deposited to public databases. We think EMBLmyGFF3 may play a role in the FAIRification of the annotation data management by helping in the interoperability of the GFF3 format.

## Limitations

As any kind of format the structure sanity of the GFF3 file used is primordial. EMBLmyGFF3 relies on the bcbio-gff library to parse the Generic Feature Format (GFF3, GFF2 and GTF), which is not dedicated to review or fix potential structure problems of the format. The EMBLmyGFF3 performs the data conversion using the current state of the mapping information present in the json files. Prior knowledge from the user about the data types contained in the GFF file and what he/she would like to be represented into the EMBL file is important in order to tune the EMBLmyGFF3 behaviour. When necessary, the users have to adjust the conversion by modifying the corresponding json files.

As the tool review each feature one by one, the computational time is tightly related to the number of features contained in the annotation file. We have seen that it could take several hours for huge genome annotation. The low speed could be inconvenient and we will work on the optimization of the computational time in a new release.
